# Cerebral Venous Thrombosis Among Bangladeshi Population: A Systematic Review

**DOI:** 10.7759/cureus.49470

**Published:** 2023-11-27

**Authors:** Redoy Ranjan, Gie Ken-Dror, Md. Atikul Aziz, Rasul Amin, Md. Shahidullah, Pankaj Sharma

**Affiliations:** 1 Biological Sciences, Royal Holloway University of London, London, GBR; 2 Cardiac Surgery, Bangabandhu Sheikh Mujib Medical University, Dhaka, BGD; 3 Physical Medicine & Rehabilitation, National Institute of Neurosciences & Hospital (NINS), Dhaka, BGD; 4 Cardiology, Bangabandhu Sheikh Mujib Medical University Hospital, Dhaka, BGD; 5 Neurology, Bangabandhu Sheikh Mujib Medical University, Dhaka, BGD

**Keywords:** cerebral venous thrombosis (cvt), venous thrombosis (ijv), cerebral venous sinus thrombosis (cvst), bangladesh, s- cerebral venous thrombosis

## Abstract

Cerebral venous thrombosis (CVT) is a rare cause of stroke which remains unsung among Bangladeshi physicians and the general population. Our objective was to provide a comprehensive review of published data on Bangladeshi CVT patients. We searched all-electronic databases for Bangladeshi studies on CVT until November 2023, including literature in all languages. This study reviews the age of onset, gender distribution, radiological characteristics, and outcomes of Bangladeshi CVT patients. We included 13 studies (two observational and 11 case reports) that evaluated 102 CVT patients and found that women suffered CVT significantly higher than men (59.8% vs 40.2%; P =0.04), respectively. The overall age of the study population was 36.6±6.8, and men were significantly older than women (45.4±12.3 vs. 32.4±8.3; P<0.001). The most commonly affected sites were the superior sagittal sinus and transverse sinus thrombosis. Rivaroxaban was primarily used for long-term anticoagulation after initial low molecular weight heparin therapy. Furthermore, most studies observed an excellent clinical outcome with completed recanalisation on early follow-up angiography in three studies. In Bangladesh, women 1.5 times more commonly suffer from CVT and 13 years earlier than men. Although this review found that prompt diagnosis and anticoagulation therapy provides good clinical outcome, we recommended further studies to evaluate the long-term outcome, especially the safety and efficacy of oral anticoagulants, with recanalisation and recurrence rate.

## Introduction and background

Cerebral venous thrombosis (CVT) is a rare cause of stroke that can lead to significant mortality and morbidity [1,2]. Its incidence has increased tenfold in recent decades due to better recognition and improved availability of advanced imaging modalities. The increased incidence in reproductive-age women is probably associated with pregnancy, puerperium, and oral contraceptives [3-5]. The landmark CVT study, International Study on Cerebral Vein and Dural Sinus Thrombosis (ISCVT) and other studies documented the occurrence of CVT in different venous sinuses: superior sagittal sinus (SSS) (45-65%), transverse sinus (TS) (41-65%), straight sinus (18%), and multiple venous sinus involvement in 46-71% cases [1-3,5].

Although CVT carries a complex pathogenesis, it is often (~50% of cases) associated with more than one risk factor, and 15% of patients are of unknown aetiology, which is why diagnosis of CVT can be challenging and requires a high index of clinical suspicion [3-5]. Additionally, chronic sinusitis and pathogen dissemination from nasal infections could be potential sources of CVT development, especially TS thrombosis, which may be caused by mastoid and middle ear infections [6,7]. Furthermore, existing literature found that female gender-specific risk factors, especially pregnancy and puerperium and oral contraceptives, are potential risk factors for CVT in women [2-4,8]. Furthermore, the American Heart Association-American Stroke Association (AHA-ASA) guidelines proposed using full-dose unfractionated or low molecular weight heparin, followed by oral anticoagulation therapy with warfarin for 6-12 months, prompt management to maximize the chance of a favourable outcome [9].

In Bangladesh, there are seldom studies on CVT, which is why there is a paucity of evidence on the prevalence, potential risk factors and prognosis of CVT following long-term anticoagulation treatment. This systematic review will focus on the age of onset, gender distribution and outcome of CVT among the Bangladeshi population to observe the age of CVT onset among sexes.

## Review

Search strategy

A literature search was conducted in the all-electronic database, including PubMed, Web of Science, Google Scholar, and BanglaJOL, for all published papers on Bangladeshi CVT of all age groups till November 2023. The Medical Subject Headings (MeSH) terms were “cerebral venous thrombosis”, “CVT”, “cerebral venous sinus thrombosis”, “CVST”, and Boolean terms were “AND” and “OR” to link search terms with for the disease.

Study eligibility criteria

**Figure 1:**
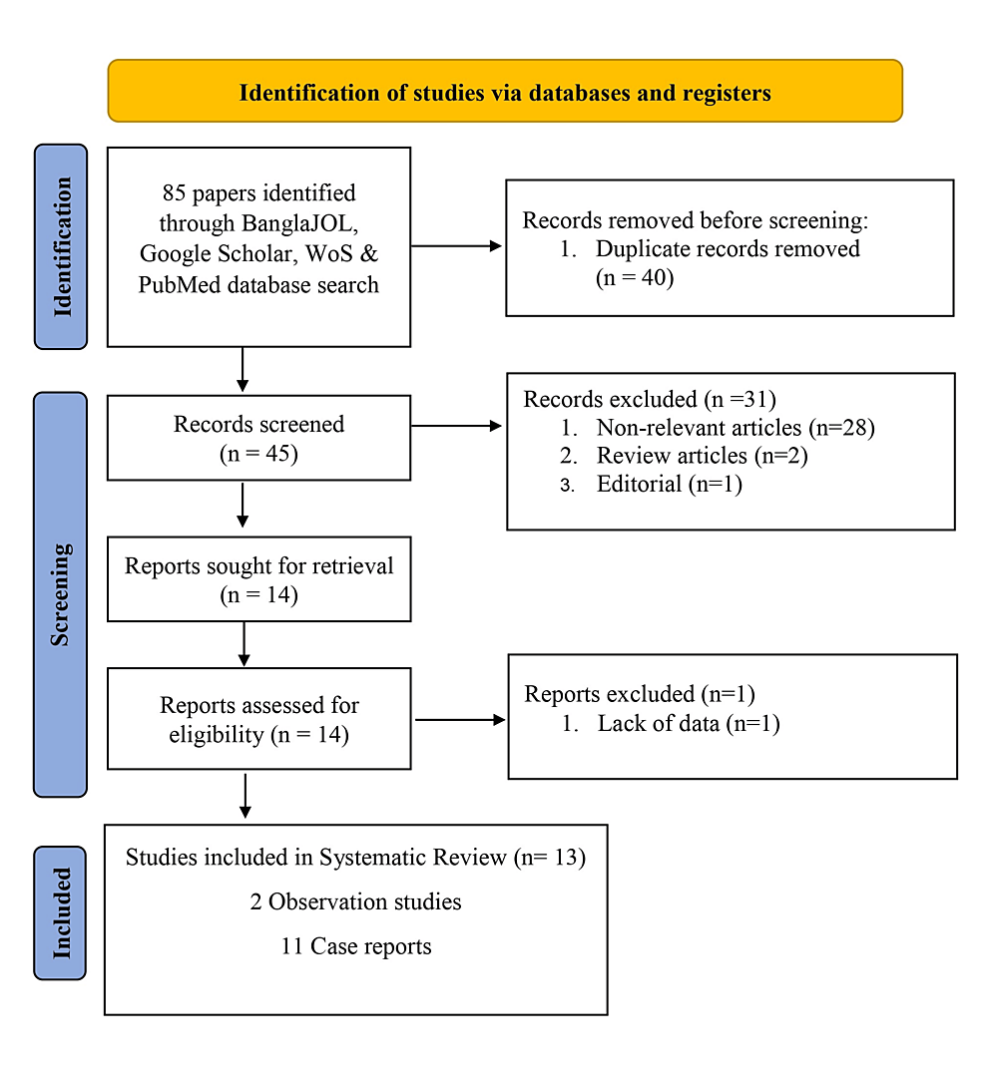
PRISMA flow diagram according to PRISMA 2020 guidelines illustrates different phases of systematic review. PRISMA: Preferred Reporting Items for Systematic Reviews and Meta-Analyses [10]

This study followed the Preferred Reporting Items for Systematic Reviews and Meta-Analyses (PRISMA) 2020 guidelines (Figure 1) [10]. The systematic review included literature on Bangladeshi CVT patients published in any language and all published studies covering case reports as well as case series on Bangladeshi CVT. However, duplicate literature was excluded from the study.

Data extraction and statistical analysis

The initial search results obtained a total of 85 existing published papers, and after careful evaluation, 13 studies (Table 1) were included in the review [11-23]. Study variables were extracted using structured data sheets and recorded into a database. A MedCalc statistical software package was utilised to calculate the age of CVT onset and gender distribution from included studies. A statistical significance was considered if a P value <0.05.

**Table 1:** Basic characteristics of the study population In Sheikh et al.’s [16] study, the female patient was 39 years old, and the male patients were 37, 42, and 73 years old. SLE: systemic lupus erythematous; APA: antiphospholipid antibody syndrome; OCP: oral contraceptive pills; MRV: magnetic resonance venography; SSS: superior sagittal sinus; TS: transverse sinus; SS: sigmoid sinus; IJV: internal jugular vein; LMWH: low molecular weight heparin; DVT: deep vein thrombosis; MDT: multidisciplinary team; HTN: hypertension.

Authors	Study type	Sample	Age; years	Women	Risk factors	Diagnostic test & Findings	Anticoagulation	Outcome	Follow-up angiography	Concluding remarks
Rahman et al.; 2023 [11]	Case-control	50	37.7	28	-	D-dimer; 526.7±97.3ng/ml	-	-	-	D-dimer can be a reliable tool for CVT diagnosis
Islam et al.; 2023 [12]	Prospective	38	28.4	24	Pregnancy, Puerperium, OCP	MRV; SSS (53%), TS (74%), SS (42%), & multiple sinus (40%)	-	-	-	Provoked CVT and TS thrombosis is more common.
Rahman et al.; 2022 [13]	Case report	1	16	1	SLE, APA	MRV; thrombosis in SSS, TS, & SS	Enoxaparin followed by Rivaroxaban	Good	-	Clinicians should be aware of recurrence with APA syndrome and continue long-term anticoagulation
Rahman et al.; 2021 [14]	Case report	1	42	1	Covid 19, Obese	CT scan; thrombosis in SSS	Enoxaparin followed by Rivaroxaban	Good	-	Early management improve outcome
Sultana et al.; 2009 [15]	Case report	1	40	1	Oral pill	MRV; thrombosis in SSS & TS	LMWH followed by warfarin	Good	Complete recanalisation	Long term oral pill in women strong predictors of CVT
Sheikh et al.; 2017 [16]	Case series	4	47.8	1	HTN	MRV; thrombosis in SSS & TS	LMWH followed by Rivaroxaban	Good	-	Rivaroxaban carries good safety and efficacy
Saha et al.; 2015 [17]	Case report	1	40	-	DM	MRV; thrombosis in SSS	LMWH	Died	-	High degree clinical suspicion required for prompt CVT diagnosis
Zaman et al.; 2019 [18]	Case report	1	22	1	Norethisterone	MRV; thrombosis in SSS	LMWH followed by Rivaroxaban	Good	Complete recanalisation at 1 month	Rivaroxaban carries good safety and efficacy in CVT
Hussain et al.; 2016 [19]	Case report	1	35	-	Hyperhomocysteinemia	MRV; thrombosis in SSS, TS, & SS	LMWH followed by Rivaroxaban	Good	-	Hyperhomocysteinemia associated with CVT
Hakim et al.; 2021 [20]	Case report	1	40	1	Polycythemia vera, Oral pill	MRV shows thrombosis in SSS, TS, & SS	Oral anticoagulant	Good	-	CVT patient may have undetected Polycythemia Vera
Fatema et al.; 2019 [21]	Case report	1	10	1	-	MRV shows thrombosis in SSS, TS & IJV	Rivaroxaban	Good	-	Combination of steroid and rivaroxaban have promising effects in CVT
Sayeed et al.; 2023 [22]	Case report	1	51	1	DVT	MRV; thrombosis in TS, SS & IJV	LMWH followed by Warfarin	Good		CVT with prior history of DVT should be screened for thrombophilia
Akhter et al.; 2019 [23]	Case report	1	32	1	Pregnancy	MRV; thrombosis in SSS	LMWH	Good	Complete recanalisation at 7.5 months	LMWH in CVT is safe and effective; MDT management is vital

Results

Age of CVT Onset

This systematic review included 13 published literature (two observational studies and 11 case reports) that evaluated a total of 102 Bangladeshi CVT patients aged between 10 years and 73 years. The overall age of CVT onset was 36.6±6.8 [11-23]. However, the age of CVT onset was significant (P <0.001) between men and women; for men it was 45.4±12.3 years, whereas women’s age was 32.4±8.3 years [13-18,20-23]. We also found that the prevalence of CVT was significantly higher among women in comparison to men [12-23] (59.8% vs 40.2%, P = 0.04).

Radiological Characteristics of CVT

Out of 13 papers, 11 studies utilised magnetic resonance venography (MRV) to diagnose CVT, and the most commonly affected sites were SSS and TS [12-23]. However, Islam and colleagues observed the highest frequency of thrombosis in TS (74%), followed by SSS (53%), sigmoid sinus (SS) (42%), and multiple sinuses (40%) [12]. The involvement of multiple sinus thrombosis was also reported in other published papers [13,15,16,19-22].

Anticoagulation Therapy

The majority of the papers mentioned initial low molecular weight heparin (LMWH) followed by oral anticoagulation therapy with rivaroxaban except for Sultana et al. and Sayeed et al. where long-term warfarin was used [13-16,18-19,21-22]. However, Akhter and colleagues diagnosed CVT during pregnancy and anticoagulation was maintained using LMWH throughout pregnancy with a good prognosis [23].

Outcome

The majority of the published papers observed good clinical outcomes except in one study where Saha and colleagues stated patient mortality was due to early discharge against medical advice [13-18-23]. Although most of the studies observed a good prognosis, only three studies reported completed recanalisation on follow-up angiography [15,18,23].

Discussion

This study revealed that women have a higher likelihood (1.5 times) of experiencing CVT than men, and they tend to suffer from it about 13 years earlier than men. The SSS and TS are the most commonly affected sinuses. Additionally, we observed that long-term anticoagulation treatment with either warfarin or rivaroxaban is safe and effective.

This systematic review highlights a paucity of data on CVT among Bangladeshi patients, indicating the need for further research to raise awareness among Bangladeshi clinicians and patients. Although we observed that women are significantly younger than men, similar to previous studies, the age of CVT onset in Bangladeshi women is earlier than in European women (32.4 vs 37 years, respectively) [24-27]. Furthermore, Ranjan and coworkers found that European women suffered CVT three times more than men, whereas Bangladeshi women suffered CVT 1.5 times more commonly than men, results similar to other South Asian studies [25-28]. However, the comparatively low CVT prevalence among Bangladeshi women might be due to lower socioeconomic status and off-limits healthcare facilities.

Although the exact pathophysiology is still unclear, several published papers observed that gender-specific risk factors (especially pregnancy, puerperium, and oral pill) are potential predictors of women's CVT, a result of concordance with the current study findings [4,24,29,30]. Nonetheless, like our review findings, Christiansen et al. and Zuurbier et al. found obesity and Martinelli et al. found hyperhomocysteinemia is a potential risk factor for adult CVT [8,31-32]. The CVT diagnosis required a high degree of clinical suspicion confirmed by angiography, either computed tomography (CT) or magnetic resonance imaging (MRI) venography [3,28]. Several published papers reported the most common involvement of SSS and TS thrombosis, similar to our findings [3,5,25-26]. Most of our studies utilised MR venography to diagnose CVT, which is helpful in either emergency or subacute cases to confirm the deep venous thrombosis and had better sensitivity and specificity over CT venography [28,33-34].

The existing treatment guidelines provided by the American Heart Association-American Stroke Association (AHA-ASA) and European Stroke Organization (ESO) recommended initial systemic heparinisation using either unfractionated or LMWH, followed by long-term oral anticoagulation with warfarin [9,35]. Despite the AHA-ASA/ESO recommendation, direct oral anticoagulant (DOAC) “rivaroxaban” was used in most studies as a long-term anticoagulation therapy due to convenient use and lower drug/food interactions, as supported by recent literature [36-38]. Although this systematic review didn’t observe any long-term evidence of anticoagulation complications from the included studies, existing literature found major bleeding including intracranial haemorrhage, non-recanalisations, and recurrence of venous thrombosis events are similar between DOACs and warfarin-treated CVT patients [11-26,28,34]. Long-term oral anticoagulation is usually recommended for 6-12 months; longer duration may be required, like CVT in antiphospholipid antibody syndrome and inheriting thrombophilia where patients require continued life-long anticoagulation because of a higher plausibility of recurrence [35-37,39]. Nonetheless, Fatema and colleagues suggest using steroids with rivaroxaban provides better outcomes, which contradicts the relevant existing guidelines, as neither ESO nor AHA-ASA guidelines support using glucocorticoids for either intracranial hypertension and cerebral oedema [9,21,35]. Finally, the treatment outcome of CVT is usually favourable, with ~85% of the patients making a complete recovery, similar to our review findings [2,9,25-26,36].

Strength and limitations

There is seldom a study on CVT in Bangladesh, which is why this systematic review generates substantial evidence based on published papers among Bangladeshi CVT patients. Further, we first describe the differences in the age of CVT onset between men and women and the prevalence of CVT among Bangladeshis. Like any study, we need to acknowledge limitations, specifically the inclusion of case reports and observational studies in the review, which are potential for bias. However, the lack of studies due to the rare CVT disease mitigates the risk of outcome bias. Although follow-up angiographic evaluation of CVT might be a concern, the low socioeconomic conditions and good clinical profile on follow-up minimise the risk of outcome bias.

## Conclusions

This systematic review observed that Bangladeshi women are significantly younger and suffer CVT 1.5 times higher than men. Considering the rarity of CVT and the paucity of enough power evidence on Bangladeshi CVT, this systematic review findings provide substantial evidence regarding the diagnosis and management of CVT. However, we recommended large-scale studies to evaluate the long-term outcome, especially the safety and efficacy of the anticoagulants, recanalisation, and recurrence of CVT.
